# Fretful Children

**Published:** 1873-09

**Authors:** 


### FRETFUL CHILDREN.

When an infant cries, and whines, and frets, and worries a great deal, grateful relief will often be found in a warm bath, placing the body in the water up to the chin. The temperature should be from ninety to a hundred Fahrenheit; that of the room should be at least seventy-five, and the duration of the bath about fifteen minutes; but it is better to measure it by the effects. As soon as the child becomes quiet, and is disposed to be playful, it should be taken out, wiped dry quickly, and wrapped up in a warm blanket for ten minutes and then dressed. Except during teething, when this warm bath may be resorted to twice a day, the cause of the fretting is almost always in the stomach; it is indigestion—the result, in almost all cases, of injudicious feeding. If no anodynes, or soothing syrups, or other forms of medicine are administered, and the child is fed at regular hours, with intervals of four or more, according to age, three fourths of all the crying of babies would be prevented.

Half of all the infants who die owe their premature decease to irregular eating and druggery.

